# Reemerging Schmallenberg Virus Infections, Germany, 2012 

**DOI:** 10.3201/eid1903.121324

**Published:** 2013-03

**Authors:** Franz J. Conraths, Doris Kämer, Kathrin Teske, Bernd Hoffmann, Thomas C. Mettenleiter, Martin Beer

**Affiliations:** Author affiliations: Friedrich-Loeffler-Institut, Greifswald-Insel-Riems, Germany

**Keywords:** Schmallenberg virus, reemergence, Germany, viruses

**To the Editor:** In 2011, Schmallenberg virus, a novel orthobunyavirus of the Simbu serogroup, emerged in Germany and the Netherlands and spread rapidly over large parts of central and western Europe (*1*–*5*). The infection primarily affects ruminants but affects camelids as well (*1*,*6*). So far, evidence has not shown that humans are susceptible to Schmallenberg virus infection (*7*). Although the infection in adult animals causes only mild symptoms (*1*) or remains clinically inapparent, in pregnant animals, transplacental transmission during a limited period can lead to the birth of severely malformed progeny (*1*,*2*). Acute infections of adult ruminants or malformed Schmallenberg virus–positive offspring have been detected on >5,000 farms in Austria, Belgium, Denmark, Finland, France, Ireland, Germany, Italy, Luxembourg, Norway, Poland, Spain, Sweden, Switzerland, the Netherlands, and the United Kingdom. Also, a high proportion of adult ruminants were seropositive for antigens of the virus in the core region affected by Schmallenberg virus in the Netherlands, Germany, and Belgium (*2*,*4*,*5*). Schmallenberg virus caused the first known outbreak of an infection with a virus of the Simbu serogroup in Europe. Schmallenberg virus infections are notifiable in Germany. Biting midges seem to play a key role in the transmission of the infection (*8*), and this transmission led to seasonal spread of the infection in summer and autumn 2011.

We report the recurrence of Schmallenberg virus infection in adult cattle, sheep, and a goat in Germany in 2012. Veterinary authorities at the county or town level report the animal holdings where laboratory-confirmed Schmallenberg virus infections are found to the central national database for notifiable animal diseases (Tierseuchennachrichtensystem), which is maintained by the Friedrich-Loeffler-Institut; the reports are made online. This database was analyzed for reported holdings with Schmallenberg virus infections that had been detected in adult animals from June 1, 2002 through October 31, 2012, and confirmed by PCR (*9*) or virus isolation. In total, 82 infections were reported in adult cattle, 8 in adult sheep, and 1 in a goat (Figure). Forty-five of the cattle holdings and 4 sheep holdings submitted samples for testing because the affected animals had shown clinical signs. One case was detected in a sheep flock, and 5 cases were detected in cattle in trade examinations. For the remaining cases, no specific reason for testing was reported.

**Figure:**
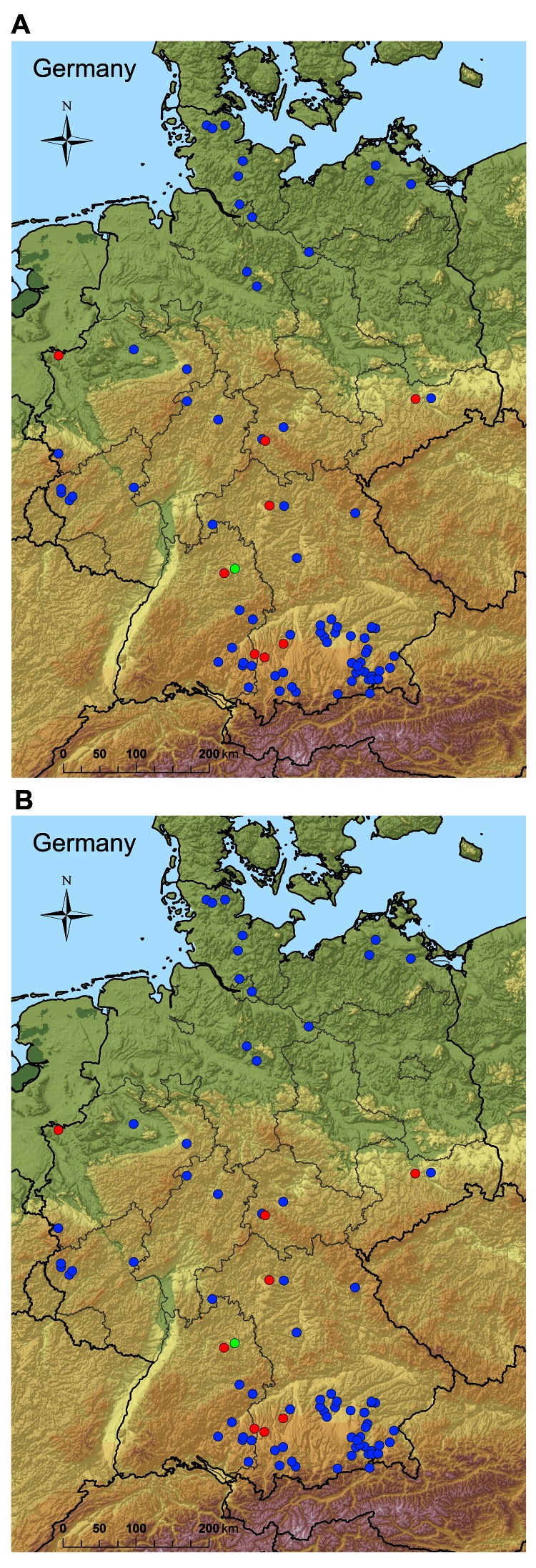
PCR-confirmed cases of Schmallenberg virus infections in Germany in A) cattle (blue dots, 791), sheep (red dots, 860), and goat holdings (green dots, 47) from August 1, 2011, through May 31, 2012; and B) cattle (blue dots, 82), sheep (red dots, 8), and goat holdings (green dot, 1) from June, 1, 2012, through October, 31, 2012.

Although some cases were reported from the region in western and northern Germany where the epidemic had its center in 2011 (Figure, panel A), several new infections occurred in regions in southern Germany where no cases or only few cases of Schmallenberg virus infection had been detected before (Figure, panel B). This phenomenon may have occurred because of a high level of protective immunity at the population level in the region affected before transmission resumed in 2012, although a substantial proportion of the animals at the margin of the affected area remained susceptible. Schmallenberg virus that has overwintered in these areas may thus be transmitted to naive animals and has apparently spread to regions in southern Germany that were not affected or were less affected by the previous Schmallenberg virus epidemic. Schmallenberg virus could also be introduced into neighboring countries through infected arthropods. Although the respective reports may not have been formally published, indications were that Schmallenberg virus had spread at least to Austria, Ireland, Finland, Norway, Poland, Sweden, and Switzerland by summer/autumn 2012.

Schmallenberg virus infection is often mild or clinically inapparent in adult animals and leads only to a short viremic period of ≈4–5 days (*1*). Because a substantial proportion of new infections in adult animals are likely not recognized, the new cases reported in Germany starting in June 2012 probably represent only the so-called tip of the iceberg. Nevertheless, PCR analysis to detect Schmallenberg virus in samples from animals with clinical signs is a valuable method for identifying first cases in areas where Schmallenberg virus infections have not previously been found. 
